# The Effectiveness of Endovenous Radiofrequency Ablation Application in Varicose Vein Diseases of the Lower Extremity

**DOI:** 10.7759/cureus.7640

**Published:** 2020-04-11

**Authors:** Fehimcan Sevil, Abdurrahim Colak, Münacettin Ceviz, Uğur Kaya, Necip Becit

**Affiliations:** 1 Cardiovascular Surgery, Afyon Health Sciences University, Afyon, TUR; 2 Cardiovascular Surgery, Ataturk University School of Medicine, Erzurum, TUR; 3 Cardiovascular Surgery, Afyon Medical Sciences University, Afyon, TUR

**Keywords:** occlusion, radiofrequency ablation, varicose vein

## Abstract

We aimed to determine the outcome, complications, and quality of life effects of radiofrequency ablation (RFA) in the treatment of superficial venous insufficiency.

A total of 134 extremities from 100 patients were evaluated in this retrospective study performed at the Cardiovascular Surgery Department of Atatürk University Faculty of Medicine. Treatment success was determined by occlusion. The clinical, etiologic, anatomic, and pathophysiologic (CEAP) and venous clinical severity score (VCSS) scores of patients were assessed pre- and postoperatively to evaluate clinical outcome and quality of life. The pain was assessed with the Wong-Baker score. Complications and their frequency were assessed and recorded.

Treatment success, as measured by occlusion rate, was 99% percent. Prior to treatment, the CEAP clinical score was C2 (81.0%), while after treatment, it was C0 (54.0%) (p<0.001). The pretreatment median VCSS score was 5 (min-max: 1-9) while the post-treatment median was 1 (min-max: 1-3) (p<0.001). The mean pain score was 1.34; only one patient reported a score of 6 while the minimum score was 1. A total of 15 complications occurred; only one was a major complication (deep vein thrombosis or DVT) while the remaining 14 were minor complications.

While longstanding surgical treatments still provide significant success, the RFA technique not only surpasses them in success rate but also in terms of pain, complications, and better patient satisfaction. The results of our study indicate that RFA is an effective and safe option for the treatment of superficial venous insufficiency.

## Introduction and background

Varicose veins are defined as enlarged and swollen veins and are quite common, especially in the lower extremities. Their frequency increases with age and they may present with symptoms ranging from cosmetic concerns to serious clinical problems. Surgical treatment options have existed for a long time; however, less invasive endovenous treatment methods have recently emerged and are reported to have superior results in terms of clinical outcome and patient satisfaction when compared to surgery [1]. Depending on definitions and the population studied, the frequency of chronic vein abnormalities are reported in the range of 30%-50% while the frequency of varices is 10%-25% [2-4]. However, the prevalence rates of chronic venous disease and varices differ greatly according to the population studied [5]. Although often regarded as a cosmetic problem by the public, varicose veins may cause symptoms such as leg heaviness, fatigue, pain, itching, swelling, burning sensation, ulcers, and restless leg syndrome, which, in many cases, result in restriction of daily activity and loss of labor.

**Figure 1:**
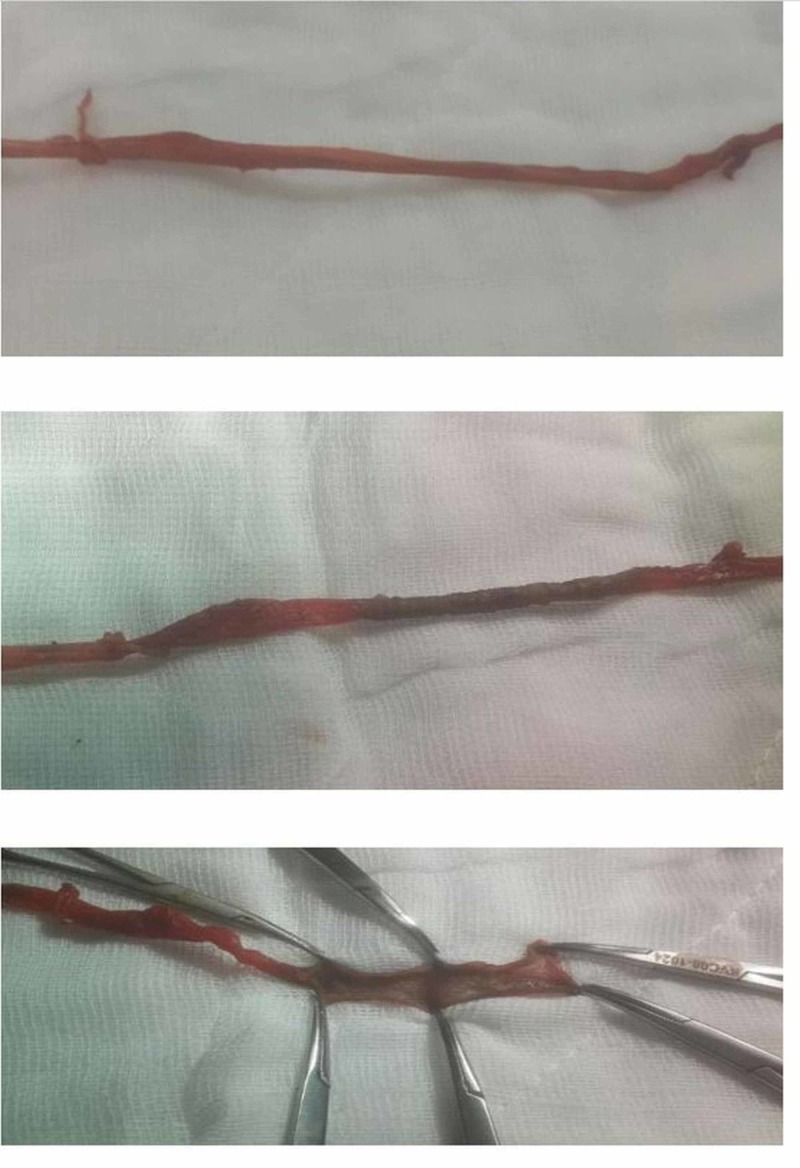
View of the saphenous vein after RF ablation RF: radiofrequency

In recent years, endovenous thermal ablation techniques have emerged as safe and effective alternatives to longstanding surgical treatments in varices associated with the greater saphenous vein. The endovascular radiofrequency ablation (EVRA) technique, also called radiofrequency ablation (RFA), is a frequently used endovenous ablation technique that achieves occlusion by the application of heat to the vein wall via a catheter that is percutaneously introduced into the lumen of the vein (Figure 1) [6]. Data from meta-analyses have shown that endovenous ablation techniques are at least as effective and safe as classical surgery while being superior in terms of complications, pain, and ecchymosis development, time until return to normal daily life activity, and patient satisfaction [7].

The studies in this field, especially those that report the clinical and quality of life (QoL) outcomes of RFA, are few and some of these do not meet the standards we have come to expect. Therefore, we aimed to determine the outcome, complications, and QoL effects of RFA in the treatment of superficial venous insufficiency.

Clinical classification

C0: no visible or palpable signs

C1: telangiectasias (veins less than <1 mm), reticular veins (1-3 mm in diameter)

C2: varicose veins (>3 mm)

C3: edema

C4: secondary skin alterations

 C4a: pigmentation, eczema, or both

 C4b: lipodermatosclerosis, white atrophy, or both

C5: healed ulcer

C6: open ulcer (often in the ankle area)

## Review

Ethical approval

The study was approved by the Clinical Research Ethical Committee of Atatürk University Faculty of Medicine. We adhered to all aspects of the Helsinki Declaration and Good Clinical Practice guidelines during the study.

Material and methods

Overview

This was a retrospective study performed by the evaluation of 134 extremities from 100 patients who underwent RFA treatment for superficial venous insufficiency from June 2013 to August 2016 at Atatürk University Faculty of Medicine, Department of Cardiovascular Surgery.

Patient Selection

Patients who had ≥3.5 mm varices of the greater saphenous vein with reflux equal to or higher than 0.5 seconds were included in the study.

The decision for RFA treatment was based on the clinical, etiologic, anatomic, and pathophysiologic (CEAP) clinical classification. Treatment was applied to those who were evaluated as C2-C6 according to CEAP as well as to patients who had significant symptoms or cosmetic concerns. The venous clinical severity score (VCSS) is a widely used instrument for evaluating venous insufficiency. It is calculated by the classification of the severity of 10 parameters (pain, varices, venous edema, pigmentation, inflammation, induration, ulcer presence, ulcer duration, ulcer size, and compliance with compression therapy). The VCSS of all patients was calculated and recorded before and after the procedure.

Patients who were under 18 years of age at the time of treatment, those who had undergone amputation in the treated limb, those in which Doppler ultrasonography (US) could not be performed (due to the limb being in a cast or similar reasons), and patients who were immobile or required assistance in daily activity for reasons other than varices were excluded from the study.

We contacted patients who had undergone RFA treatment between June 2013 and August 2016 by phone and proposed participation in the study. A clinical visit was scheduled with those who accepted and patients were re-informed about the study during the face-to-face meeting. All patients provided informed consent for the study. Treatment success was determined by venous occlusion.

Treatment Procedure

**Figure 2:**
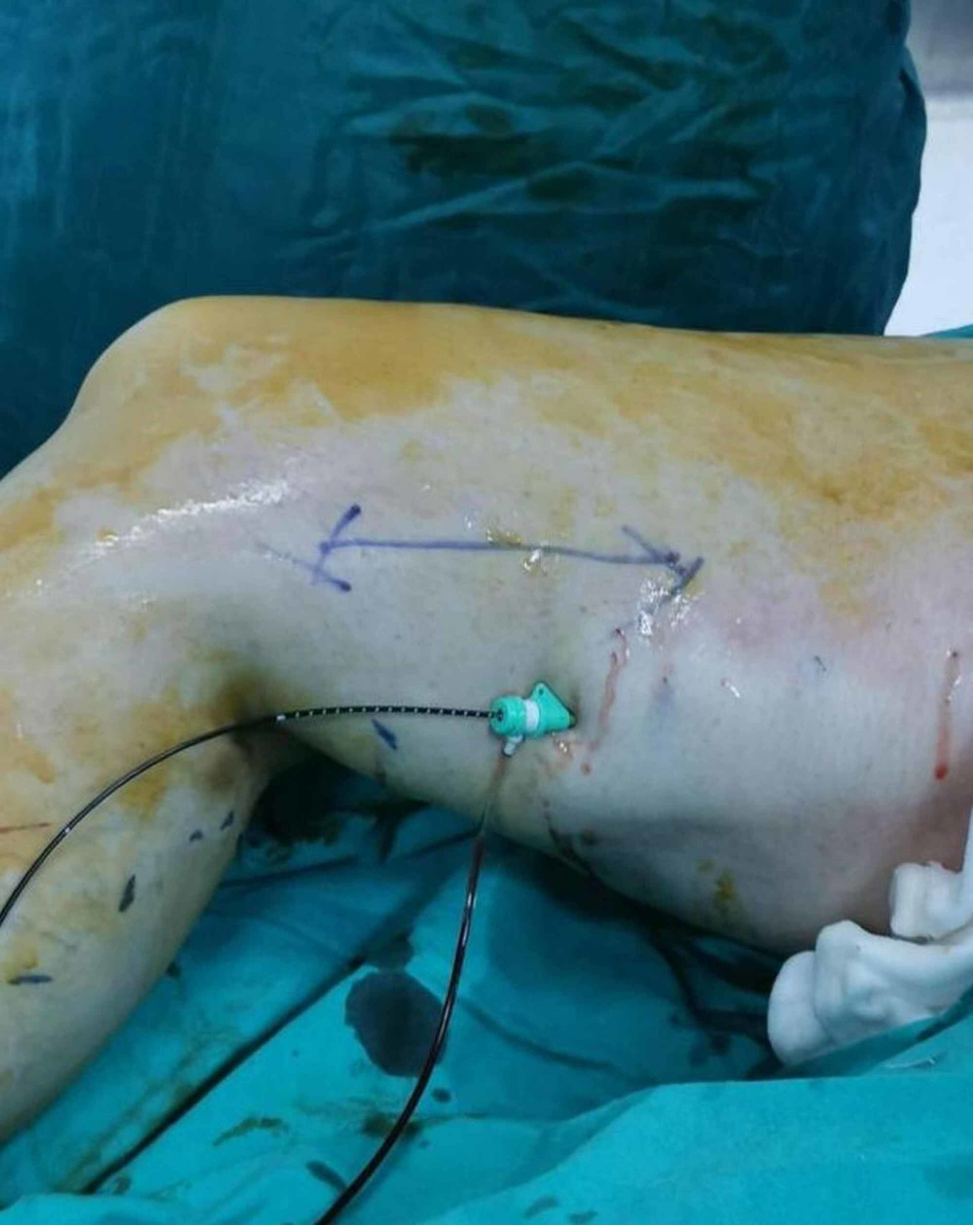
Placed view of the catheter during the operation

**Figure 3:**
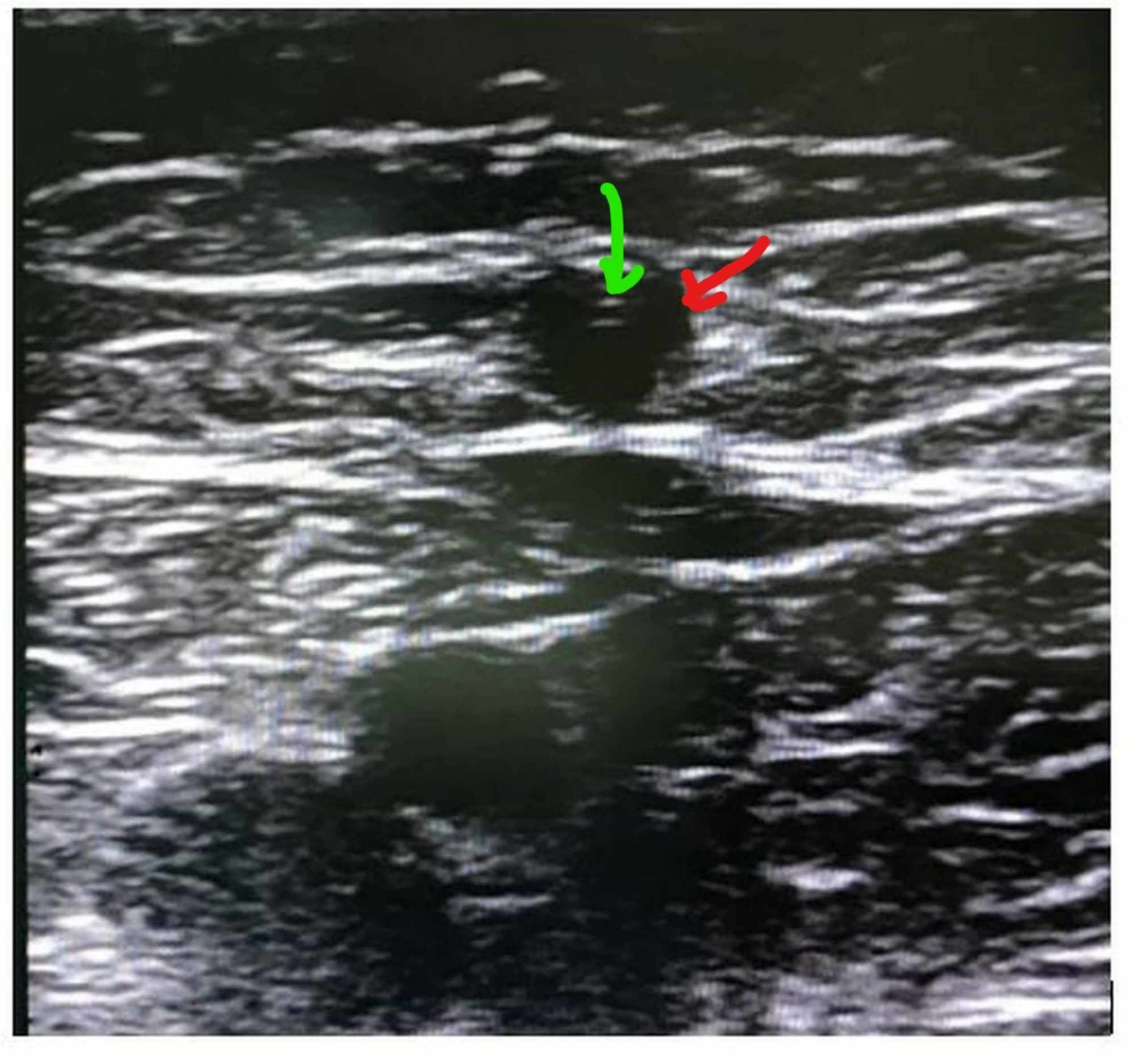
Image of RFA catheter in VSM Red point: great saphenous vein; Green point: RFA catheter RFA: radiofrequency ablation

All procedures had been performed in sterile operating room conditions. A brief explanation of the procedure is as follows: The site of intervention (dilated veins and the greater saphenous vein) were marked before the procedure. After the application of spinal anesthesia, patients were put into the supine reverse Trendelenburg position. Following disinfection with povidone-iodine (Betadine) solution, a 6F sheath was inserted into the greater saphenous vein from an appropriate position above the knee with Doppler USG guidance via the Seldinger technique (Figure 2). An RFA catheter was advanced until we reached 2 cm distal to the saphenofemoral junction (SFJ) and the position was checked with Doppler USG (Figure 3). Before ablation, varicose dilatations were excised with the mini phlebectomy technique. Then, a solution containing 20 ml 2% lidocaine, 10 ml 8.4% sodium bicarbonate, and 0.5 mg adrenalin in 500 cc's 500 cc serum physiologic was applied into the saphenous compartment with a 21G needle. RFA was performed with external pressure on the greater saphenous vein while the patient was in the Trendelenburg position. After the procedure, the elastic bandage was applied to the extremity for two to five days. The patients were given compression socks (with 20-30 mmHg pressure) for six months.

Post-treatment and Follow-up Evaluations

**Figure 4:**
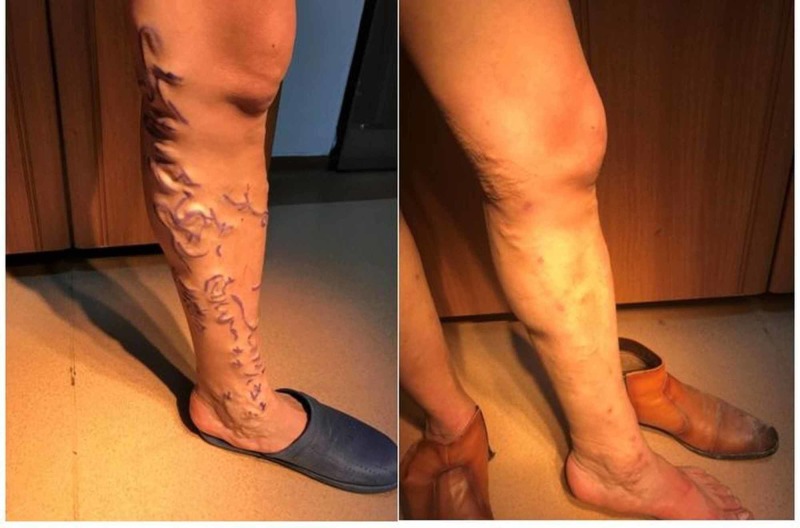
Pre and postoperative view

Follow-up studies comprised a physical examination and Doppler US. They were planned and conducted after the procedure at post-treatment Day 1, at the end of the first week, and the first, sixth, twelfth, and twenty-fourth months (Figure 4). The procedure-related pain of patients was assessed with the Wong-Baker visual pain score. All patients were evaluated with pain score, without discharge. Post-interventional complications, including ecchymosis, hyperemia, induration, skin-burn, paresthesia, hematoma, and edema, were recorded. To determine the presence of reflux and/or DVT at the SFJ and other factors, such as occlusion and recanalization, Doppler USG was performed while the patient was in the upright position. The VCSS and CEAP clinical stages were calculated to determine treatment success and symptomatic recovery. During all follow-up studies, the VCSS and CEAP clinical stage of patients and the presence of pain, paresthesia, ecchymosis, edema, hematoma, and DVT were treatment. Doppler US investigations were also performed and the Union Internationale de Phlebologie (UIP) classification system was used for the assessment of reflux at the SFJ and occlusion and/or recanalization of the greater saphenous vein.

Statistical Analysis

All analyses were performed on SPSS Statistics version 21.0 (IBM, Armonk, NY) for Windows. The normality of distribution was checked with the Shapiro Wilk test. Data were given as mean ± standard deviation (SD) for normally distributed data and median (minimum-maximum) for non-normally distributed data. Categorical variables were given as frequency (percentage). For the comparison of pre- and post-interventional scores, the Wilcoxon signed-rank test was used for continuous data and the marginal homogeneity test for categorical data. p ≤ 0.05 values were accepted as significant.

Results

We included 100 patients (64 males and 36 females) in our study. The mean age was 41.47 ± 11.71 years. Thirty-four patients had bilateral venous insufficiency so the total number of extremities that underwent the procedure was 134. The most common symptoms before treatment were pain (57.0%), swelling (50.0%), and fatigue (46.0%). Venous diameters varied within a range of 4.5-17 mm. The most common anesthesia method was spinal anesthesia (100%) (Table 1). The pain levels of all patients were evaluated with the Wong-Baker visual scoring system and the highest score was 6 (one patient), the lowest score was 1, and the mean score was 1.34. 

**Table 1:** Descriptive variables of the study group

	N	Value
Age		41.47 ± 11.71
Sex		
Male	64	64.0%
Female	36	36.0%
Body Mass Index	59	26.77 ± 3.24
Effected Side		
Right	28	28.0%
Left	38	38.0%
Bilateral	34	34.0%
Symptoms		
Pain	57	57.0%
Swelling	50	50.0%
Pruritus	32	32.0%
Fatigue	46	46.0%
Cramp	34	34.0%
Venous Diameter	134	8.00 (4.50 - 17.00)
Anaesthesia		
spinal	100	100%
Wong-Baker Visual Pain Score		
0	45	45.0%
1	0	0.0%
2	44	44.0%
3	0	0.0%
4	10	10.0%
5	0	0.0%
6	1	1.0%
SFJ Reflux	15	15.0%
Venous Reflux	14	14.0%

All patients were ambulated at two hours after the procedure. Seventy-seven patients were discharged by post-treatment Day 2 while all patients had been discharged by Day 6 (inclusively). Seventy-two patients returned to normal daily activity within one week and all patients within 10 days (inclusively). VCSS was found to be significantly decreased after treatment (p<0.001). The most common CEAP clinical score was C2 (81.0%) before treatment and C0 (54.0%) after treatment (p<0.001). The pre-treatment median VCSS value was 5 (min-max: 1-9) and the post-treatment median value was 1 (min-max: 1-3). The difference was statistically significant (p<0.001) (Table 2).

**Table 2:** Pre- and post-treatment comparisons regarding the VCSS and CEAP classifications VCSS: venous clinical severity score; CEAP: clinical, etiologic, anatomic, and pathophysiologic

	Pre-treatment		Post-treatment		P	
VCSS [median (min-max)]	5 (1 - 9)		1 (1 - 3)		<0.001	
CEAP Clinical Classification	N	(%)	N	(%)		
C0	0	0.0%	54	54.0%	<0.001	
C1	0	0.0%	44	44.0%		
C2	81	81.0%	2	2.0%		
C3	12	12.0%	0	0.0%		
C4	4	4.0%	0	0.0%		
C5	3	3.0%	0	0.0%		
C6	0	0.0%	0	0.0%		

Complications developed after 15 of the procedures. The most common complication was bleeding (n=5). One of our patients developed DVT (Table 3). None of the patients underwent repeat treatment because none had further complaints.

**Table 3:** Complications and their frequency (percentage)

Complication	N	Percentage	
Ecchymosis	2	2.0%	
Hematoma	2	2.0%	
Thrombophlebitis	3	3.0%	
Bleeding	5	5.0%	
Paresthesia	2	2.0%	
Deep Venous Thrombosis	1	1.0%	

Discussion

In the current study, the clinical outcome of varicose vein treatment with RFA was investigated through the pre- and post-treatment evaluation of VCSS and CEAP classification, which showed significant improvements in both parameters. Regarding daily activity, all patients who underwent RFA returned to normal daily activity within 10 days of the procedure. Pain scores were also very low for the large majority of patients, the mean score was 1.34 and only one patient reported a score of 6.

Today, the endovenous laser ablation (EVLA) and RFA techniques are the most popular endovenous thermal ablation techniques. The EVLA technique utilizes laser for energy delivery while the RFA technique is based on energy delivery via radiofrequency. These techniques provide important advantages as compared to classical surgery and the literature on this topic has reported lower recurrence rates after the emergence of endovenous ablation techniques [7]. The RFA technique applies heat via electrodes that are in direct contact with the lumen. This thermal impact causes endothelial damage, which is ensued by venous spasms and collagen destruction, resulting in endovenous obliteration. This acute effect is followed by the activation of collagen remodeling and fibroblast proliferation, both of which contribute to lumen obstruction [8]. Data from a meta-analysis that collectively evaluated 12,320 lower extremities from 119 studies showed that the percentage of success was 78% with stripping treatment, 77% with foam sclerotherapy, 84% with RFA, and 94% with EVLA [7]. Several studies that investigated occlusion rates with endovenous ablation techniques have reported similar occlusion rates with RFA and EVLA (90%-100% and 95%-100%, respectively [9-10]. Likewise, the percentage of success with RFA was 99% in the current study. Several other studies have reported success rates ranging between 94.5% and 96% with RFA treatment [11-13].

Studies on this topic unanimously report that, regardless of treatment method, the outcome is very favorable and venous symptoms are almost completely resolved with treatment [14-16]. Although outcomes are ultimately similar, various studies have shown better perioperative findings with RFA when compared with classical surgery [17-19]. In terms of outcome measures, RFA treatment was found to provide significant improvements as seen by the improvement in CEAP and VCSS scores of our patients. In a study by Choi et al., which also utilized RFA, the CEAP clinical stage had improved 2.33 ± 0.78 to 1.29 ± 0.96 and VCSS score had improved 3.48 ± 0.98 to 0.63 ± 1.16 [13]. Two other studies also reported significant improvements in both scores, especially VCSS [11-12]. Pain, either immediately after the procedure or in the long run, is a very important factor when the patient’s satisfaction is considered. Several studies have reported lesser pain with RFA than with classical surgery and laser treatment [20-23].

In the current study, a total of 15 complications occurred. However, only one patient developed a major complication (DVT). Other major complications (pulmonary embolism, skin burn, infection and nerve damage) did not occur. A few studies have suggested that DVT may be an important complication for RFA treatment with reported frequencies from 0.2% up to 16% [24]. The Medical Advisory Secretariat (MAS) review found that major complications had developed in 105 patients among the 3,664 patients who underwent RFA treatment. Major complication rates, in reducing frequency, were as follows: DVT, infection, skin burns, nerve damage, and pulmonary embolism [25]. In other studies, only Zuniga et al. and Marsh et al. reported DVT complications (3.5% and 0.7%, respectively [26-27]. Pulmonary embolism was also reported in only two studies with low rates of occurrence (0.04%, 1.4%) [26,28]. When compared with laser treatment, skin burns were reported to be less frequent in patients who underwent RFA (1.3% vs. 2.6%), which shows another advantage of RFA [22]. Minor complications, such as swelling, bruising, hematoma formation, and development of inflammation and paresthesia, can be considered quite common after RFA treatment [20-21]. Minor complications that developed in the current study and their frequency were as follows: ecchymosis in two, hematoma in two, thrombophlebitis in three, minor bleeding in three, and paresthesia in two patients.

Considering the available literature and our findings, RFA therapy may be considered better than other treatment options, especially regarding the reduction in pain and frequency of complications as compared to laser and classical surgery. However, although a minor complication, paresthesia has been reported to be more frequent with RFA. In the current study, only two patients had paresthesia; however multi-center studies with a higher number of patients are necessary to determine actual frequencies of minor complications in RFA, as the level of evidence in available studies is low.

Our study has several limitations. First, the number of patients in this single-center study is low as compared to several large-scale and multicenter studies. Second, the lack of a control group (for quality of life comparison) and a comparison group that underwent another treatment (to compare outcomes) is a limitation. However, forming a control group without treatment would be unethical; therefore, the lack of this group was unavoidable. Additionally, as we aimed to report the findings and results of RFA treatment, a comparison group that underwent other treatments was not formed; further studies that compare the findings of various treatments are required.

## Conclusions

Superficial venous insufficiency is a condition that adversely affects the QoL of patients. Although longstanding surgical options have existed for quite some time, endovenous ablation techniques have emerged as treatments that are equally effective and safe while also being minimally invasive. The RFA technique that we have explored in our study is an effective and safe treatment that provides lower complication rates and increases patient satisfaction during the peri- and postoperative periods.
